# Psychological Capital and Entrepreneurship Sustainability

**DOI:** 10.3389/fpsyg.2020.00866

**Published:** 2020-05-19

**Authors:** Jun-Jun Tang

**Affiliations:** College of Economics and Administration, Nanjing University of Aeronautics and Astronautics, Nanjing, China

**Keywords:** psychological capital, entrepreneurship sustainability, future research, perspective, review

## Abstract

Successful formation of a new venture is not the most critical indicator of the real success of an entrepreneurial venture. Instead, the sustainability of an entrepreneurial venture (i.e., entrepreneurial sustainability) is the most critical but the most difficulty goal. Entrepreneurial sustainability relies largely on positive collective psychology. This article offers systematic and detailed discussion of the effects of psychological capital on the critical elements of entrepreneurship sustainability – not just that on a successful formation of a new venture. The goal is to shed light on potential practical designs and theoretical improvement in the future.

## Background

The psychological capitals are generally found to have immense contribution toward providing competitive advantage of a firm since it is difficult to imitate by the competitors. The Psychological Capital (commonly referred as PsyCap) are generally found to influence behavior and attitude of employees which, in turn, has a direct impact over improving organizational performance (Newman et al., 2014). This is because PsyCap determines the emotional intelligence of an employee and enables them to be motivated by staying unaffected due to negative consequences. Hence, it enables an individual to foster innovation and creativity that will be able to boost organizational performance. A clear understanding about Psychological Capital will enable in improving individual as well as organizational performance. Moreover, the positive Psychological Capital helps to inculcate a motivating and supportive work culture thereby enabling the firm to stay ahead of its competition. This will help to design work as individuals with higher motivation are expected to efficiently achieve organizational goals.

According to Avey et al. (2010), the four core constructs of PsyCap are efficacy, hope, optimism and resilience, which are directly related to attitudes and emotions of employees that then might affect their behavior or behavioral intentions. It is believed that a healthy work environment creates a good feeling among employees and motivates them to achieve higher degree of success. Moreover, Psychological Capital is directly linked with well-being of individuals which, in turn, will be reflected in terms of their performance. In other words, Psychological Capital is concerned with bringing improvement within an individual both personally and professionally. The occupational health has been found to be directly associated with physical well-being of individuals. Thus, individuals prefer to work with organizations having positive work environment which, in turn, enables a firm to retain a pool of competent employees.

The positive psychological state of an employee provides the confidence to take certain degree of risks generally referred to as self-efficacy. It also enables a person to reconstruct paths for achieving its goals and encourages to sustain in a challenging situation. These factors depict that psychological capital is positively related with the improvement of performance at a particular job role. Peterson et al. (2011) state that psychological capital is manifested through motivation levels, enhancement of cognitive capabilities and capability to pursue goals. All the above stated attributes enable an individual to achieve a higher degree of success by bringing constant improvement in business performances.

In such vein, this commentary article sets to discuss the potential links between PsyCap and a sustainable entrepreneurship journey of entrepreneurs and his/her entrepreneurial team(s). This topic at the intersection of the two important research fields is important but has been under-researched. Through this reflective piece of work, hopefully more future studies suitably fall into the intersection of PsyCap and entrepreneurship, especially in a more sustainable, dynamic time frame, could be stimulated to offer theoretical and practical implications (Luthans et al., 2007; Matzler and Renzl, 2007; Hofstede, 2011; Owoyemi et al., 2011; Ahmad et al., 2014; Anitha, 2014; Paterson et al., 2014; Saxena, 2014; Youssef-Morgan and Luthans, 2015; Petrides et al., 2016; Walker et al., 2016).

## Psychological Capital and Sustainable Entrepreneurship

According to Fakhri et al. (2012), motivational and cognitive excellence is the key force which stimulates entrepreneurial capabilities within an individual. The success of an entrepreneurial journey lies in the ability to identify opportunities and take necessary actions for overcoming all challenges faced in the entire process, which requires both cognitive and motivational strengthen of an entrepreneur. The psychological construct within an individual are generally developed during the infant stage and are mostly influenced by the environment in which a person is brought up. It can be clearly stated that entrepreneurs who are positively influenced personally tend to achieve a greater degree of success. In other words, personal traits mostly influence the entrepreneurial capabilities of an individual.

Thus, the presence of a positive psychological capital has been found to facilitate entrepreneurial development of an individual. The basic qualities which define an entrepreneur include the ability to take risks, capability to facilitate innovation, capability to lead a particular team and many more (Langkamp Bolton and Lane, 2012). These attributes enable a firm to participate in the innovative culture and benefit the organization as a whole.

Overall, the discussions below could be depicted into the conceptual framework to improve comprehensiveness.

First, the sustainability of entrepreneurial activities is an indication of stronger entrepreneurial intentions which also depicts their exploratory capabilities. Therefore, the relation between personal characteristics and creative process enables a researcher to understand how psychological capital can stimulate continuous creativity generation, which is a foundation of entrepreneurship, within an individual. According to Sweetman et al. (2011), creativity is the capability of an individual to introduce new ideas that will bring modification in terms of products, services and processes such that it improves the overall performance of the organization. Hence, creativity can be considered to be a key for achieving organizational competitiveness and survive in the competitive environment. The psychological capital of an employee motivates to enhance cognitive bases which, in turn, will facilitate creative thinking. The willingness to take risks associated with an innovative process enables an individual to formulate strategies and explore various alternatives. Hope is one among the four attributes associated with psychological capital which enables an individual to stay optimistic. This enables an individual to take risks and succeed in an innovative process. It is imperative that the ability to think positively depends on personal attributes which helps to keep a control over negative situations. Thus, positive psychological capital has been found to facilitate creativity generation and re-generation of an individual and associate a greater degree of sustainable success.

Second, leadership is critical in sustaining entrepreneurial development. A sustainable entrepreneur with higher leading capability could ensure better economic stability for a new venture to successfully navigate complex situations without being defeated by internal conflict, inefficient coordination, and heterogeneous interests and motivation of all partners. The organizations are generally found to promote sustainable entrepreneurship by motivating experienced personnel by leadership to identify the opportunities available in external business environment and benefit the firm as a whole. Further, the culture of an organization is enforced by leaders as the capability of leading and directing a particular group of people depends on the efficiency of leadership. Hence, a sustainable entrepreneur will be able to motivate subordinates for following sustainable practices that will ensure successful achievement of organizational goals.

Third, sustainable entrepreneurs are generally found to maintain a balance between economic, social and environmental factors which in turn helps to safeguard interests of every stakeholder (Kuckertz and Wagner, 2010). This will enable the firm to sustain in the competitive environment by choosing the course of actions which will be beneficial in the long term. A sustainable entrepreneur has the capability of driving business processes in a socially responsible manner such that the practices undertaken by the firm does not leave a negative impact over the environment (DiVito and Bohnsack, 2017). Those entrepreneurs intend to generate profits by considering various environmental factors. All of these imperatives require entrepreneurs’ good orchestrating of their stakeholders in the surrounding environment. The sustainable entrepreneurship is an important topic in an organizational context since it enables a firm to sustain in the competitive environment. Shepherd and Patzelt (2011), states that sustainable entrepreneurship is concerned with preserving both the environment and society in which it operates such that it continues to provide opportunities in future times. Hence, it is imperative that sustainable entrepreneurs tend to select the paths that will be aligned with the stakeholder objectives and ensure long term stability for the firm.

Psychological capital of the entrepreneurs and his/her entrepreneurial team could help communication and coordination with the stakeholders. Those traits of optimism, hope and resilience help entrepreneurs to retain communication and coordination even when there are obstacles and disagreements. Moreover, the trait of self-efficacy, whether at individual- or group-levels, could enable entrepreneurs to communicate and coordinate in a mode confident and “charming” way.

Fourth, PsyCap facilitates entrepreneurs’ capability of facing dramatic changes, which might lead to extremely positive or negative outcomes. An entrepreneurial environment constantly undergoes innumerable changes in terms of social, political, economic and technological factors. Under such circumstances, human talent such as an entrepreneur plays an important role in making business firms adaptable to the changing environment. This will enable the firm to continue its operations for a long period of time by ensuring socio-economic development of the entire society from which the firm procures resources. With PsyCap the employees have the capability of increasing efficiency and making a particular organization effective, it is the responsibility of management (i.e., the entrepreneurs here) to ensure a proper utilization of such human resource (i.e., the employees in new ventures). A proper human resource management practice will enable a firm to attract and retain competent employees and make them behave in a socially responsible manner (Araque et al., 2018), for which PsyCap could do a great help.

The growth and development of business firms are directly related with social welfare. This is because firms are responsible for creating jobs for the community, and therefore, ensure its economic development through equal income distribution. This depicts that business firms depend on the society for necessary resources including human and psychological capitals. The business firms, in return, has certain responsibilities toward the society that provides necessary resources for continuing its operations. The firms will have to construct the operations in a sustainable manner such that it does not leave any detrimental impact over the environment. It is imperative that entrepreneurship and sustainability can be combined together for developing a new approach in ensuring social welfare.

Last but not least, PsyCap facilitates entrepreneurial innovation that leads to more sustainable entrepreneurship. Innovation enables an individual to make optimal utilization of resources and thus plays a crucial role in ensuring sustainable development. This is achieved since it establishes appropriate price-cost structure which, in turn, enables a firm to manage processes in a cost-efficient manner. The benefits offered by sustainable practices are generally found to be beneficial in safeguarding environment and thus, are preferred by most of the consumers in recent times. In this competitive business environment, firms adopting innovative sustainable practices are found to achieve a distinguished position in the market. The strategic thinking and decision making capabilities of an entrepreneur have been found to effective in designing processes that will prove to be productive for the firm as a whole (Welsh and Krueger, 2012). The entrepreneurial abilities enable to identify opportunities and convert them into a valuable resource with the application of appropriate techniques. This is because entrepreneurs possess the capability of viewing a particular aspect from multiple perspective and integrate innovation for developing a method profitable for the firm as a whole.

Psychological capital enables an individual to develop creative ideas and thus is responsible for fostering innovation within a particular firm. Positive psychological capital can, therefore, be considered to be a manifestation of personality traits since it is concerned with delivering value to an organization through the set of skills possessed by an individual. It is the ability of an individual to effectively utilize knowledge and manage them by forming social networks. The four major constructs of psychological capital can be explained to depict how it facilitates in the entrepreneurial journey. Self-efficacy can be referred to as the confidence toward achievement of a particular goal. It, therefore, enables an individual to continue with a particular course of action by overcoming all possible challenges. Hope, on the other hand, signifies the positive energy that motivates an individual to explore all possible opportunities and achieve a distinguished position among competitors. Optimism, however, enables an individual to expect positive outcome for the processes undertaken and bring greater degree of success for the firm. Resilience enables a person to have a control over emotional stability which will help to overcome challenges in difficult situations (Ziyae et al., 2015). The attributes associated with psychological capital, therefore, facilitate entrepreneurial sustainability within an organization.

The business firm in this competitive environment strives to find innovative ways that will enable them to achieve a distinguished position among others. This has made them depend on the innovative ideas possessed by human resources that will enable the firm to sustain for a long period of time. In other words, organizations in order to survive in the competitive environment need to recruit innovation led persons such that it facilitates in implementing a progressive work culture. It is equally important for the management to ensure that employees do not feel stressed as it hinders innovative capabilities. To ensure entrepreneurship sustainability, the management therefore need to concentrate on factors that will provide satisfaction toward the employees (Abbas and Raja, 2015). On the other hand, individuals having higher psychological capital tend to have less stress related to job. This, in turn, depicts that personal attributes are directly related with stress and anxiety experienced by a person. It has also been found that employees having higher degree of psychological capital exhibit more innovative nature such that it proves to be productive for the organization. It can be stated that people with higher degree of psychological capital can contribute toward sustainable development of the organization through its entrepreneurial skills.

## Commentary

The above section has illustrated the findings of peer-reviewed journals related to psychological capital and entrepreneurial sustainability. The discussion is concerned with emphasizing that psychological capital improves entrepreneurial sustainability from an organizational perspective. The analysis presented above reveals that psychological capital has direct impact over organizational performance. psychological capital is characterized by four major attributes namely self-efficacy, hope, optimism and resilience. Self-efficacy refers to the confidence within an individual for achieving a common objective and thus, provides the confidence of overcoming all possible challenges. Hope represents the energy with which an individual feels motivated to explore all possible opportunities. Optimism, implies the positive attitude of an individual which enables to perceive about positive outcome of a particular happening. Resilience enables a person to hold a control over the emotional quotient and manage complex situations effectively. The above stated attributes are completely in accordance with the qualities to be possessed by an entrepreneur. This provides definition for an entrepreneur which refers to the individual capable of introducing new ideas that will eventually enable the firm to achieve a distinguished position among competitors. This implies that the individual needs to be confident enough for overcoming all challenges in order to succeed in the course of action.

An entrepreneur needs to be positive regarding the outcomes of a particular situation that will enable it to effectively manage complex situations. Under such circumstances, it is essential for the individual to hold stability in terms of emotional state that will facilitate in the decision making process. The psychological capital refers to the emotional intelligence which motivates an individual to stay motivated toward achievement of a particular objective. The positive psychological capital within employees, therefore, benefits in creating a participative work culture. This creates motivation within the firm and enhances performance as a whole. The discussion has also stated that psychological capital is related with the well-being of employees. Thus, psychological capital promotes innovation which is a major attribute of an entrepreneur.

It has been found in the above analysis that psychological capital is related to personal traits of an individual and is influenced by the surrounding environment in which the individual is brought up. Thus, individuals who are personally motivated toward achieving a particular goal tend to achieve greater degree of success. The individuals having a positive attitude toward work can prove to be beneficial for an organization thereby depicting the presence of entrepreneurial skills. Sustainable entrepreneurship refers to the strong intentions present within an individual for exploring all possible opportunities. Such practices provide greater degree of success to an organization by keeping a proper balance between external and internal business environment. It is also evident that management needs to concentrate on effectively coordinating human resources such that psychological capital proves to be beneficial for the firm. The utilization of psychological capital is appropriate when it creates a positive environment resulting in personal development and enforcement of capabilities for dealing with challenging situations, and prepares a person to be determinant about a desired outcome and ability to proceed further by overcoming all possible challenges (Cavus and Gokcen, 2015). This reflects that psychological capital has an immense contribution toward developing sustainable growth within an organization.

Psychological capital can be measured and effectively controlled by the application of proper resources such that it increases performance as a whole. The success of an organization, thus, depends on the psychological capital available within an organization and also on the participation from every employee. It sets the right attitude toward work and also influences others which enables a firm to achieve long term success. In other words, psychological capital implies mostly the positive aspect of an individual behavior. Hence, it signifies hope, creativity, wisdom, responsibility and other aspects of human nature which are necessary for becoming an entrepreneur. These positive aspects of a human nature are generally found to overshadow the negative aspects such as stress and mental exhaustion. It, therefore, concentrates on bringing a positive transformation within an individual by realizing future state from its present condition.

It can be clearly stated from the above analysis that psychological capital is related with cognitive capability of an individual and thus, has been found to be correlated with innovative capability of an individual. Sustainability in entrepreneurship are generally found to create a balance between economic, social and environmental factors that will prove to be beneficial for a long period of time. Thus, it depends on the innovative capability of an individual which enables to identify opportunities and select the correct course of action. Moreover, the cognitive capabilities enable an individual to associate the proper resources such that it facilitates in the introduction of new improved processes. The above discussion suggests that a sustainable entrepreneur has the capability of constructing processes in a socially responsible manner which can only be possible due the presence of positive psychological capital.

The four constructs of psychological capital reveal that it is directly related with entrepreneurial skills and are directly related with personal trait of an individual. Conversely, presence of positive psychological capital is generally found to enhance health and well-being of individuals. In addition to that, sustainable entrepreneurship is generally found to resolve some of the vital social as well as environmental concerns. Thus, sustainability in entrepreneurship are expected to be a growing concern among consumers and organizations following sustainable practices are more preferred over others. It is clear from the analysis that in recent times, firms are found to adopt sustainable practices for gaining competitive advantage and gain higher degree of profitability. This is the major reason that most of the firms show preference toward adopting sustainable practices and consider psychological capital as a means of integrating it into business processes.

The management, in recent times, have been found to concentrate on recruiting individuals on the basis of positive psychological capital. Along with selecting appropriate human capital, it is equally important for the management to nourish those talents by providing them with a supportive environment. This signifies the importance of psychological capital in developing a positive culture within an organization and also proves its influence on improving its performance. A good work environment creates a positive attitude within employees and motivates them to perform better. The individuals thereby working under a supportive environment are likely to be less stressed. This again proves that psychological capital is directly related with well-being of employees. Furthermore, psychological capital has been proved to be dependent on personal traits. The individuals who have been brought up in a positive environment are likely to possess positive psychological capital. The individuals are likely to be beneficial for the firm due to their innovative nature.

The above discussion has also explained the qualities of a sustainable entrepreneur as such practices are generally found to ensure better economic stability for an organization. The positive psychological capital is generally found to enable entrepreneurs to undertake sustainable practices by exploring all possible opportunities. The ability of an individual to positively evaluate the outcome which is an important construct of psychological capital and facilitate entrepreneurship. Sustainable entrepreneurship has evolved since it has enabled the firms to adopt cost-efficient processes that will also be able to preserve environment. The socio-economic benefits provided by the sustainable entrepreneurship has made firms to promote these practices as it ensures better stability in the long term. These practices result in the development of the society in which an organization operates and relies heavily for procuring useful resources.

The above discussion is intended to depict contribution of psychological capital in positively influencing sustainable entrepreneurship. It can be clearly stated from the above discussion that psychological capital promotes health and well-being of individuals. Apart from this, it leaves a positive impact over organizational culture and motivates others for actively participating in various processes. This, in turn, highlights that positive psychological capital promotes innovation which is an important skill possessed by entrepreneurs. A positive relation has been noticed between psychological capital and sustainable entrepreneurship as individuals having higher degree of PsyCap are generally found to be considerably affected by work-related stress. Hence, those individuals are expected to be of good well-being which in turn is reflected through their performance. It is evident that psychological capital has a significant contribution toward improving organizational performance.

The positive psychological construct of an individual enables to take risks and sustain in a challenging situation through emotional stability. The basic constructs of psychological capital therefore, highlight its interrelation with entrepreneurship. The positive psychological capital enables an entrepreneur to think strategically and introduce new ideas within an organization. The sustainable entrepreneurs are generally found to concentrate on integrating processes that will be economical as well as prove to be beneficial for the environment. The practices have been found to result in socio-economic development of the society and ensure long term performance of the firm. The business firms procure resources from the society which in turn compel to carry on certain responsibilities toward it. The sustainable practices in recent times have been found to gain attention of majority of population. This has encouraged business firms to adopt such practices and emphasize on developing innovative ideas that will prove to be beneficial for the firm in the long term.

It can be clearly stated from the above analysis that sustainability in entrepreneurship has been found to be benefit the firm in long term and incur better financial results. The business firms, in order to achieve competitive advantage and gain a distinguished position in the market, tend to adopt sustainable practices for ensuring long term stability of a firm. This has encouraged them to rely mostly on psychological capital that will enable to continuously bring innovation in its processes. The positive psychological capital enables an entrepreneur to be self-motivated to continue with innovative practices. Moreover, an individual having higher psychological capital tend to be more productive for the firm and depict to possess better health condition. This enables them to improve productivity as such individuals has less absenteeism, motivated toward their job and many more. Hence, all these attributes facilitate creativity stemmed from positive psychological construct and benefit the organization as a whole.

The above discussion explained on the basis of extracts derived from peer-reviewed journals has narrated that sustainable entrepreneurship is positively related to psychological capital and also varies on the basis of personal characteristics. Employees working within an organization belong from diverse background and their motivation level tend to vary. Thus, it is the responsibility of management to enforce a positive culture within the organization that will improve overall productivity. The management also needs to concentrate on recruiting individuals having positive psychological capital that will facilitate in developing a motivating culture. This practice has been found to be conducive for an entrepreneur as attitude of every employee will be positively influenced. It will be convenient for entrepreneurs to design training programs on the basis of psychological capital that will in turn enhance knowledge base and improve productivity of the organization as a whole (Smith and Woodworth, 2012). The interaction of entrepreneurs with its subordinates have been found to have significant impact over psychological capital and attitude of employees.

It can be inferred from the above analysis that degree of psychological capital affects performance of firms. Moreover, the environment in which employees work are generally found to affect its psychological capital. This highlights the importance for an entrepreneur to understand various organizational situations and focus on managing a cordial relation with subordinates. This will encourage employees to share their opinions and facilitate the innovative process. The attitude of employees depicts the degree of association of an individual with job and consequently provides a brief idea regarding individual performance. It can be concluded that in recent times, firms have been found to concentrate on sustainable entrepreneurship which is positively related with psychological capital of an individual for achieving long terms stability.

## Conclusion

According to Kyrö (2015), entrepreneurs refer to those individuals having the capability of introducing new ideas through individual efforts such that it results in economic development for the organization and society at large. The entrepreneurs need to rely on the raw ingredients abundantly available in the nature. This necessitates them to undertake the right course of action that will enable to preserve those resources for a long period of time. To achieve the goal, entrepreneurs need to effectively design sustainable practices for preserving natural resources by the proper application of various capital, including the PsyCap discussed in this article.

Furthermore, it has been found that firms are reliant on sustainable entrepreneurship for ensuring their long term success by exploring new opportunities available in the market. Terziev (2016) states that strategies resulting in economic, social and environmental development can be facilitated through entrepreneurship. The integration of innovative practices results in increase of productivity for the particular firm and also improves the quality of products or services delivered. The application of psychological capital enables proper construction of infrastructure which eventually enhance competitiveness of a firm. This is because entrepreneurs, by adopting a new approach toward planning of the production process, enable a firm to improve productivity in a socially responsible manner. Sustainable entrepreneurship has been found to improve health standards of individuals which also includes safeguarding biological cycles. Thus sustainable entrepreneurship is considered to be a panacea for a range of emerging social and environmental concerns.

## Author Contributions

The author confirms being the sole contributor of this work and has approved it for publication.

## Conflict of Interest

The author declares that the research was conducted in the absence of any commercial or financial relationships that could be construed as a potential conflict of interest.
