# Correction: Psychological capital and entrepreneurship sustainability

**DOI:** 10.3389/fpsyg.2025.1693510

**Published:** 2025-09-18

**Authors:** Jun-Jun Tang

**Affiliations:** ^1^College of Economics and Administration, Nanjing University of Aeronautics and Astronautics, Nanjing, China; ^2^Huaiyin Institute of Technology, Huaian, China

**Keywords:** psychological capital, entrepreneurship sustainability, future research, perspective, review

Affiliation Huaiyin Institute of Technology was erroneously omitted for author *Jun-Jun Tang*.

The original version of this article has been updated.

